# Unexpected Role of Physiological Estrogen in Acute Stress-Induced Memory Deficits

**DOI:** 10.1523/JNEUROSCI.2146-20.2020

**Published:** 2021-01-27

**Authors:** Rachael E. Hokenson, Annabel K. Short, Yuncai Chen, Aidan L. Pham, Emily T. Adams, Jessica L. Bolton, Vivek Swarup, Christine M. Gall, Tallie Z. Baram

**Affiliations:** ^1^Departments of Anatomy and Neurobiology; ^2^Pediatrics; ^3^Neurobiology and Behavior; ^4^Neurology, University of California-Irvine, Irvine, California 92697

**Keywords:** estrogen, hippocampus, memory, sex differences, stress, synapses

## Abstract

Stress may promote emotional and cognitive disturbances, which differ by sex. Adverse outcomes, including memory disturbances, are typically observed following chronic stress, but are now being recognized also after short events, including mass shootings, assault, or natural disasters, events that consist of concurrent multiple acute stresses (MAS). Prior work has established profound and enduring effects of MAS on memory in males. Here we examined the effects of MAS on female mice and probed the role of hormonal fluctuations during the estrous cycle on MAS-induced memory problems and the underlying brain network and cellular mechanisms. Female mice were impacted by MAS in an estrous cycle-dependent manner: MAS impaired hippocampus-dependent spatial memory in early-proestrous mice, characterized by high levels of estradiol, whereas memory of mice stressed during estrus (low estradiol) was spared. As spatial memory requires an intact dorsal hippocampal CA1, we examined synaptic integrity in mice stressed at different cycle phases and found a congruence of dendritic spine density and spatial memory deficits, with reduced spine density only in mice stressed during high estradiol cycle phases. Assessing MAS-induced activation of brain networks interconnected with hippocampus, we identified differential estrous cycle-dependent activation of memory- and stress-related regions, including the amygdala. Network analyses of the cross-correlation of *fos* expression among these regions uncovered functional connectivity that differentiated impaired mice from those not impaired by MAS. In conclusion, the estrous cycle modulates the impact of MAS on spatial memory, and fluctuating physiological levels of sex hormones may contribute to this effect.

**SIGNIFICANCE STATEMENT:** Effects of stress on brain functions, including memory, are profound and sex-dependent. Acute stressors occurring simultaneously result in spatial memory impairments in males, but effects on females are unknown. Here we identified estrous cycle-dependent effects of such stresses on memory in females. Surprisingly, females with higher physiological estradiol experienced stress-induced memory impairment and a loss of underlying synapses. Memory- and stress-responsive brain regions interconnected with hippocampus were differentially activated across high and low estradiol mice, and predicted memory impairment. Thus, at functional, network, and cellular levels, physiological estradiol influences the effects of stress on memory in females, providing insight into mechanisms of prominent sex differences in stress-related memory disorders, such as post-traumatic stress disorder.

## Introduction

Chronic stress (lasting days to weeks) disrupts hippocampus-dependent spatial memory (Sunanda et al., 2000; Kleen et al., 2006). Conversely, acute stress (lasting minutes to hours) can enhance memory and promote survival (Sandi et al., 1997; Uysal et al., 2012; Brivio et al., 2020). Surprisingly, we have previously discovered that, as opposed to a single acute stress, several short stressors imposed simultaneously (multiple concurrent acute stresses [MAS]) impair spatial memory in male rats and mice enduringly (Chen et al., 2010, 2016; Maras et al., 2014). This is important because such acute traumatic events, consisting of combined and simultaneous physical, emotional, and social stresses, are increasingly recognized to provoke memory-related problems, including post-traumatic stress disorder (North et al., 1994; Tempesta et al., 2012; Lowe and Galea, 2017; Musazzi et al., 2017; Novotney, 2018).

Stress and memory processes, and their interactions, differ across sexes. Males and females generally excel in different types of memory, and sex can influence the cognitive strategies an animal uses to solve a task (Qiu et al., 2013). Some of these differences are hormone-independent, whereas others are strongly influenced by the estrous cycle and associated fluctuations in the sex steroid hormones estrogen and progesterone. For hippocampus-dependent spatial memory, high estrogen levels, whether exogenous or naturally occurring, often facilitate memory (Gresack and Frick, 2006; Tuscher et al., 2019; Luine and Frankfurt, 2020), potentially by increasing synapse-bearing dendritic spines (Gould et al., 1990; Woolley et al., 1990; Vierk et al., 2014). However, high estrogen levels, whether endogenous or experimentally induced, may also worsen hippocampus-dependent memory and plasticity (Warren and Juraska, 1997; Snihur et al., 2008; Barha et al., 2010; Tanaka and Sokabe, 2013; Sabaliauskas et al., 2015). Notably, estrogen effects on hippocampal memory are highly sex-specific, with the estrogen requirement generally thought to be more pronounced in females (Vierk et al., 2012; Frick et al., 2015; Wang et al., 2018).

Sex differences are prominent in the mechanisms and consequences of stress. Compared with male rodents, females mount a greater neuroendocrine response to stress (Heck and Handa, 2019; Zuloaga et al., 2020). This response may be hormone-dependent, as higher estrogen levels are associated with greater stress responses (Viau and Meaney, 1991; Lund et al., 2006; Liu et al., 2011). Furthermore, female rodents can be affected by stresses that may have the opposite effects in males (Bowman et al., 2001; Luine, 2002; Conrad et al., 2003; Ortiz et al., 2015; Peay et al., 2020).

Memory deficits are a common and understudied component of stress-related disorders, and susceptibility can differ between sexes (Christiansen and Hansen, 2015; Olff, 2017). Therefore, it is imperative that studies probing the biological substrates of stress-related memory deficits be expanded to include females and analyzed with respect to sex hormones. We have previously shown that MAS impairs hippocampus-dependent memory and dendritic spine integrity in male mice. Here we tested whether MAS affects hippocampus-dependent memory in female mice and determined the impact of sex hormone fluctuations across the estrous cycle on protection or vulnerability to MAS. Spatial memory, assessed using two independent memory tasks, was impaired following MAS in female mice with high physiological levels of estradiol (entering proestrus), and spared in females stressed during estrus, when estradiol levels are at their nadir. Dendritic spine density in dorsal CA1, thought to be a proxy for excitatory synapses, was reduced in high estradiol females following MAS. *Fos* expression, a marker of neuronal activation, was differentially distributed in high- and low-estrogen stressed females, delineating functional networks across salient brain regions that differentiated these groups.

## Materials and Methods

All experiments were conducted according to National Institute of Health guidelines on laboratory animal welfare and approved by the Institutional Animal Care and Use Committee at the University of California-Irvine.

### 

#### Animals

Two- to 4-month-old female virgin C57BL/6J or B6.Cg-Tg(Thy1-YFP)16Jrs/J transgenic mice, expressing YFP under control of the Thy1 promoter (Thy1-YFP), were received from The Jackson Laboratory or bred in house. Mice were group-housed 2–5 mice per cage in a quiet, uncrowded facility on a 12 h light/dark cycle (lights on at 6:30 A.M.) with *ad libitum* access to water and food (Envigo Teklad, 2020x, global soy protein-free extruded). Female mice were housed with same-sex cage mates in individually ventilated cages with Envigo 7092-7097 Teklad corncob bedding and iso-BLOX nesting material. Temperature was maintained between 22°C and 24°C. The number of animals used is detailed in each respective methods subsection.

#### Estrous cycle monitoring

Estrous cycle phases were monitored daily via vaginal cytology. Briefly, a PBS-moistened small cotton-tipped applicator (Puritan 890-PC DBL) was inserted into the vagina, and the walls of the vagina were scraped for cells. These cells were then smeared across a gelatin-coated microscope slide (Fisherbrand 12-552-3). After drying, slides were stained with methylene blue using the Shandon Kwik-Diff Kit (Thermo Fisher Scientific, 9990700), and cell types were identified under a microscope to classify cycle phases (Caligioni, 2009; Byers et al., 2012). Vaginal smears were collected within the first four hours of the light cycle, except on the day of MAS (or control), where they were collected up to an hour before lights on. Cycles were monitored for at least two complete cycles before behavioral or histologic assessments. Mice were selected to be in early proestrus/high estradiol (E2) or estrus/low estradiol (E2) at the time of MAS. For cases in which the mouse was not killed on the day of MAS, estrous cycle smears were collected for at least one more day to ensure accurate cycling. Specifically, mice classified as estrus/low E2 on the day of MAS were either still in estrus or beginning metestrus by the next day depending on cycle length. Mice classified as early proestrus/high E2 for MAS were late proestrus to early estrus the next day. If these cycle classification conditions were not met, the mouse was excluded from behavioral analysis. Mice that were not cycling were not used or experiments were postponed until normal cycling was reestablished. We limited our proestrus groups to early proestrus, when estradiol levels are high and before the progesterone surge (Becker et al., 2005), although we did not measure progesterone and cannot exclude its potential effects. To quantify vaginal smear cell type composition, images of the smears were taken under 4× magnification. Cell types were manually classified by a trained observer and counted by overlaying a grid over the image through ImageJ. Cell types were expressed as percentage of smear.

#### Multiple concurrent acute stresses (MAS)

Mice from both cycle phases were assigned to the MAS group or to the home-cage control group. The MAS paradigm involves exposing mice to simultaneous physical, emotional, and social stresses. Briefly, mice were individually restrained in a ventilated 50 ml plastic tube. Two to six mice were placed in a cage atop a laboratory shaker in a room bathed with loud (90 dB) rap music and bright lights for 2 h. This protocol is described in detail at Bio-protocol (Hokenson et al., 2020) and has been used in other studies (Maras et al., 2014; Chen et al., 2016; Libovner et al., 2020). MAS started within the first 2 h of the light cycle. For behavioral assessments, mice underwent MAS for 2 h, were returned to the homeroom for 1 h, then moved to the behavioral testing suite to acclimate for 1 h before tests. For spine and *fos* experiments, mice underwent MAS for 2 h and then were immediately anesthetized for perfusions. Home-cage control (unstressed) mice were taken from their home cage, immediately injected with a lethal dose of a 1:10 dilution of Euthasol (488 mg/kg pentobarbital sodium and 63 mg/kg phenytoin sodium, intraperitoneally) in the vivarium, and transported to the laboratory for perfusion.

#### Learning and memory tests

##### Object location memory (OLM) task

**Figure 1.**
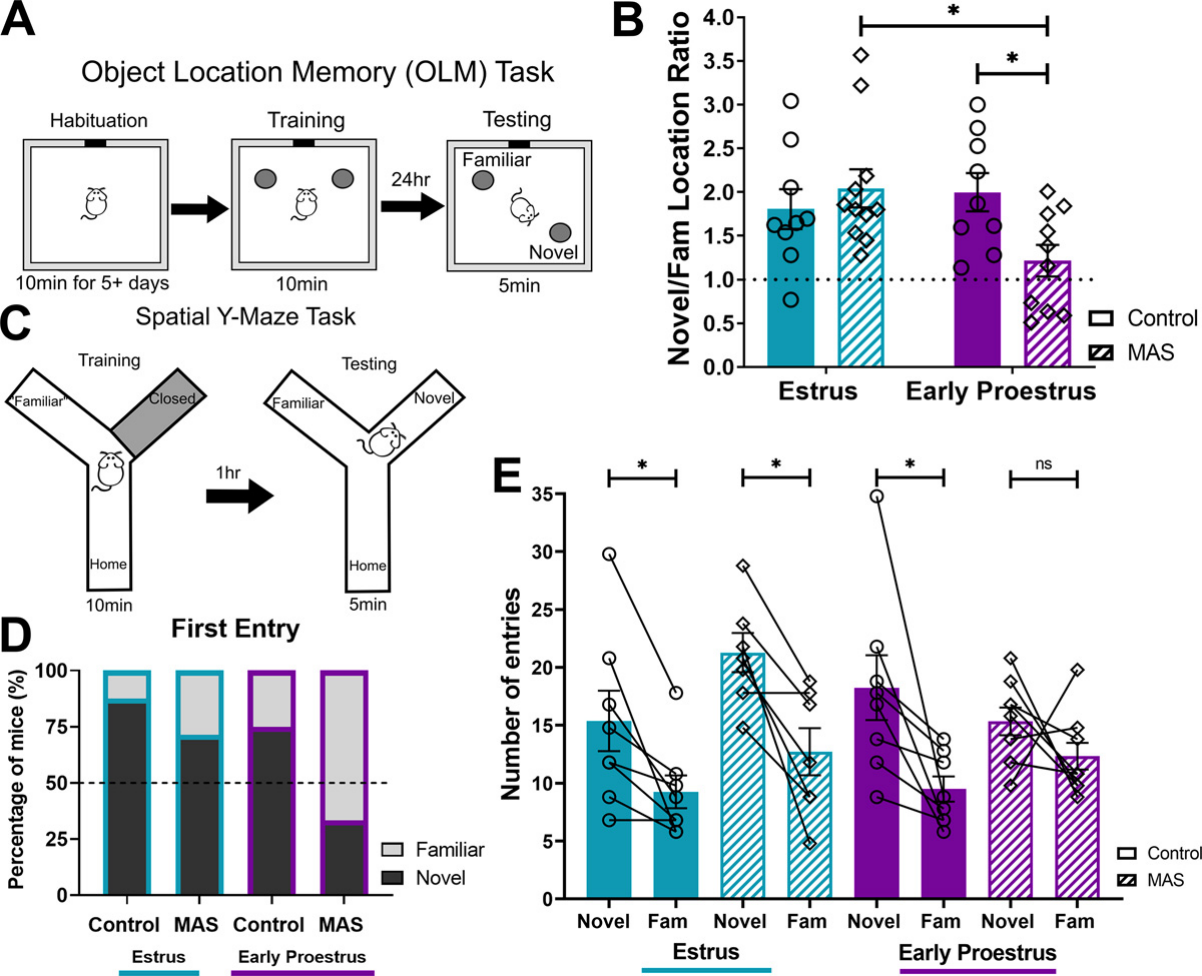
Spatial memory impairment following MAS is limited to mice entering proestrus. ***A***, For the Objection Location Memory (OLM) task, mice were habituated to the apparatus several days before MAS. At 2 h after MAS, mice were trained and then memory was tested 24 h later. ***B***, Estrus control, estrus MAS, and early proestrus control mice preferentially explored the object in a novel location, whereas early proestrus MAS mice explored both objects equivalently (*n* = 9-11/group). ***C***, For the spatial Y-maze task, 2 h after MAS, mice were trained in the apparatus with one arm closed. After 1 h, mice were reintroduced to the maze with the previously closed arm (the novel arm) now open. ***D***, Most mice of both control groups and most estrus MAS mice entered the novel arm as their first choice, whereas the first entry being the novel arm for MAS early proestrus mice was below chance. ***E***, Estrus control, estrus MAS, and early proestrus control mice entered the novel arm more frequently than the familiar arm, whereas early proestrus MAS mice entered the novel and familiar arms equally (*n* = 7–9 per group). *p < 0.05 (post-test). Points represent scores of individual animals. Connected points are matched samples within an animal. Error bars indicate ± SEM.

The OLM task is hippocampus-dependent (Vogel-Ciernia et al., 2013). OLM was performed as illustrated in Figure 1*A* (adapted from Vogel-Ciernia and Wood, 2014). Mice were handled for at least 2 min a day for at least 6 d, first in the housing room and then in the behavioral suite for the last few days. After handling, mice were habituated to an empty experimental apparatus for 10 min a day for 5–11 d. If the mouse was not in a proper cycle phase on the sixth day, habituation continued until the mouse was in an appropriate phase. In the training portion of the OLM task, two identical objects were presented to the mouse for 10 min. This training session took place 2 h after the cessation of MAS. Twenty-four hours later, one object (counterbalanced) was moved and exploration was recorded for 5 min. Object exploration was scored by observers unaware of the experimental groups using BORIS version 6 (Friard and Gamba, 2016). Investigation was defined as the mouse's nose being pointed toward the object within 1 cm distance; time climbing or biting an object was not included. Object preference was defined as the amount of time exploring the displaced object divided by time exploring the unmoved object, with a ratio of 1 indicating no preference. Total exploration time was calculated and compared across groups. Mice were excluded if they explored for <10 s total during training, <5 s total during testing, or <1 s exploration for a single object. No mice had an object bias during training (ratio <0.5 or >2.0) that would warrant exclusion. Nine to 11 mice were used per group. Four mice were excluded from analyses for under exploration (one per group), and 2 mice were excluded because of incorrect cycle determinations (one in each early proestrus group).

##### Spatial Y-maze task

The spatial Y-maze is a hippocampus-dependent task (Conrad et al., 1996; Sarnyai et al., 2000) that offers the advantage of a short training-to-testing interval, such that both are accomplished within the same day. The Y-maze was performed as illustrated in Figure 1*C* (adapted from Mo et al., 2014) in a separate cohort of mice. Mice were handled for at least 2 min a day for at least 6 d, first in the housing room and then in the behavioral suite. Distal cues were arranged around the Y-maze. In the training portion, one arm (counterbalanced) was closed off with a divider. For 10 min, the mouse was permitted to explore the home arm (the arm into which they were initially placed) and the familiar or open arm. The mouse was then returned to their cage for a 1 h intertrial interval. The divider was then removed; and in the 5 min testing phase, the mouse was permitted to explore all three arms of the Y-maze. Whether the mouse's first entry was into the novel or familiar arm during the testing phase was recorded. The number of entries into the novel arm were compared with entries into the familiar arm as an assessment of location preference. Total arm entries were calculated for the training phase (home and familiar) and the testing phase (home, familiar, and novel) to compare general exploration between groups. Furthermore, distance traveled during training and testing was used to compare general activity between groups. Video tracking software (Noldus Ethovision 15) was used to compute distance traveled and arm entries. Seven to nine mice were used per group, and no mice were excluded from these analyses.

#### Uterus dissection

Uterine indices were determined by standardizing the uterus wet weight with the animal's body weight ((uterine weight (g)/body weight (g)) × 100). The mouse was weighed, and vaginal smears were taken before death. Animals were killed via rapid decapitation (10:00 AM to 12:00 PM) and the uterus was removed. All surrounding tissues, including fallopian tubes, were removed and uterine wet weight measurements were taken. Uteri were harvested without knowledge of cycle phase, thus resulting in uneven group sizes (eight uteri were from mice in low E2 and 17 uteri were from mice in high E2). No mice were excluded from these analyses.

#### Brain processing and analyses

Analyses of dendritic spines and *fos* expression were conducted immediately after MAS or in unstressed controls. Immediately after being removed from MAS, mice were anesthetized with a lethal dose of a 1:10 dilution of Euthasol (488 mg/kg pentobarbital sodium and 63 mg/kg phenytoin sodium, intraperitoneally) and perfused intracardially with freshly prepared 4% PFA in 0.1 m sodium PB, pH 7.4, 4°C. Brains were cryoprotected and sectioned into 20 µm slices.

#### Imaging and quantification of hippocampal dendritic spines

Using the Thy1-YFP mice, which allow for clear visualization of axon terminals, neurons were chosen using systematic unbiased sampling from the dorsal hippocampus (Chen et al., 2001). CA1 pyramidal neurons were selected for analyses to include equal representation of long- and short-shaft populations. *z*-stack images were captured, reconstructed, and drawn using a Carl Zeiss 510 confocal microscope with 63× objective, ImageJ (version 2), and Adobe Photoshop (version 5). The second to fourth apical dendritic branches of CA1 pyramidal neurons were collected at 0.2 μm focal steps through the entire depth of each dendrite. Six neurons from six sections per animal, and 4 or 5 animals per group were evaluated.

The number of spines (spine density) was quantified comparing dendritic branches of the same order. Reconstructed spines were identified and characterized (Chen et al., 2013); mushroom-type and thin spines were compared (mushroom and thin spines were combined to compute total spines), and filopodia were excluded. Spine density was expressed as the number of spines per 10 μm of dendrite length. No correction factors were applied to the spine counts because high-magnification neuronal reconstruction permitted all spines of a given dendritic segment to be visualized. All analyses were performed without knowledge of treatment group, and 2 mice (1 from each high E2 group) were excluded for improper cycle categorization.

#### Imaging and *fos* expression analyses

An avidin-biotin complex, DAB reaction was used to visualize *Fos* protein in the anterior paraventricular thalamus (PVT), paraventricular nucleus of the hypothalamus (PVN), dorsal hippocampus (cornu ammonis, dentate gyrus: CA1, CA2/3, DG), amygdala (central, basolateral, medial: CeA, BLA, MeA), anterior division of the bed nucleus of the stria terminalis (BNST), and septum (lateral [LS], medial [MS]) for each mouse. Sections were washed with PBS with 0.3% Triton X-100, quenched with 0.09% H_2_O_2_, then blocked with 2% normal goat serum and 1% BSA. Sections were incubated overnight at room temperature in rabbit anti c-*Fos* primary antibody (1:10,000, Sigma Millipore, ABE457, lot #3088370), washed, then incubated for 40 min in biotinylated goat anti-rabbit IgG (1:400, Vector Laboratories, BA-1000). Sections were stained with Vectastain Elite avidin-biotin complex peroxidase kit for 3 h, stained for DAB (Vector DAB peroxidase substrate kit), then mounted and coverslipped with Permount mounting medium.

Images of sections were taken at 4× magnification (Nikon Eclipse E400, Nikon DS-Fi3, NIS-Elements F version 4.60.00). One section per region was analyzed, and borders of the entire region were delineated with reference to a mouse brain atlas (Sidman and Pierce, 1971; Paxinos and Franklin, 2001). Anterior-posterior bregma coordinates of each analyzed region were as follows: LS and MS 1.18 mm, BNST 0.38 mm, PVT and PVN −0.56 mm, MeA −1.06 mm, CeA and BLA −1.46 mm, and CA1, CA2/3, and DG −2.5 mm. The BNST regions included constitute the anterior division, including the anterolateral and anteromedial areas according to the Allen Mouse Brain Atlas (Lein et al., 2007). A single, 4× magnified image was analyzed for all regions except the dorsal hippocampus, in which two images were combined. Counts represent unilateral regions except for midline regions (PVT and septal) that were counted as a single structure (bilateral). *Fos*^+^ cells were identified in ImageJ using the analyze particle function (Schneider et al., 2012). Images were first converted to 16-bit black and white, the background was subtracted, and the hole fill feature was used. Fos staining was not dense enough to warrant any corrections for overlapping particles, and automated counts were highly correlated with manual *fos*^+^ cell identification. All analyses were performed without knowledge of treatment group. All available regions were analyzed in each mouse for 6 or 7 mice per group, and no mice were excluded from these analyses. Raw *fos* counts were scaled (mean normalization) across all brain regions and groups before computing correlation matrices. Correlation matrices were used to construct comparative network maps.

#### Estradiol ELISA

Blood serum estradiol concentration was quantified using a Mouse/Rat Estradiol ELISA kit (Calbiotech, ES180S-100). The Calbiotech kit has a 3 pg/ml functional sensitivity, 3.1% intraassay precision, and 9.9% interassay precision, as provided by the manufacturer (Haisenleder et al., 2011). Blood was collected from the mouse after rapid decapitation (9:00 AM to 11:00 AM) and clotted at room temperature for ∼30 min. Samples were centrifuged at 1100 rcf for 15 min. The clear supernatant was collected, stored at −20°C, and the pellet discarded. Samples were thawed and run in duplicates according to the manufacturer's instructions. Absorbances were read within 15 min at 450 nm with a microplate reader (BioTek Synergy HTX). Data of three separate ELISAs were analyzed together, but concentrations per sample were computed using the standard curve generated during each respective run. Estradiol was quantified in 12 low E2 and 18 high E2 mice, classified by matched vaginal smear data. One mouse from the high E2 group was excluded for poor cycling, and 1 mouse from the low E2 group was excluded because of an error on the ELISA plate. Estrogen levels in the majority of the estrus group samples fell below the 3 pg/ml sensitivity of the kit and were extrapolated. This approach enabled drawing correlations between cytology and hormone levels across the cycle phases. We also used the alternative approach, setting values under the limit at zero (undetectable). This approach also yielded robust differences in mean estradiol levels between high E2 and low E2 and vaginal cytology cell types.

#### Experimental design and statistical analyses

Statistical analyses were performed using GraphPad Prism version 8.4.2 for Windows (GraphPad software) or R, including packages: igraph, impute, qgraph, DescTools, Hmisc, and corrplot (Csardi and Nepusz, 2006; Epskamp et al., 2012; Wei and Simko, 2017; Harrell, 2020; Hastie et al., 2020; R Core Team, 2020; Signorell et al., 2020). Two- or three-way ANOVAs were used for behavioral, spine, and *fos* data when two or three factors were analyzed (factors identified in the respective Results sections). Sidak's multiple comparisons post-tests were run when a main effect or interaction was found to be statistically significant (α = 0.05) or if a specific comparison was planned (cases identified in Results). Uterine indices were compared across the two groups with an unpaired *t* test. Estradiol levels and vaginal smear cell types were found to be not normally distributed by Anderson-Darling test for normality. A nonparametric, Mann–Whitney test was used to compare estradiol levels between the two groups. Correlations between estradiol and estrous cell types were computed using nonparametric Spearman Rank-Order Correlations. Pearson product-moment correlations were also computed to generate a best fit line. Correlation matrices for regional *fos* expression were computed using Spearman Rank-Order Correlations. To compare *fos* activity networks between groups, correlation coefficients were converted to *z* scores, and the difference in *z* scores was plotted using the R package qgraph, which represents an increase or decrease in correlation (color) and intensity of the difference (line thickness). Differences between *z* scores were computed by calculating the *z*_observed_ (*Z*_obs_ = (*Z*_1_ – *Z*_2_)/(√[(1/*n*_1_ – 3)+(1/*n*_2_ – 3))), and these values are presented in Table 1. Data point exclusions are elaborated on for each section of Materials and Methods. The results are reported as mean ± SEM unless noted otherwise.

**Table 1.** Difference between correlation coefficients (*Z*_obs_) across conditions

Region	Control: high E2 vs low E2 (*Z*_obs_)	High E2: MAS vs control (*Z*_obs_)	Low E2: MAS vs control (*Z*_obs_)	MAS: high E2 vs low E2 (*Z*_obs_)
CeA and BNST	0.61	0.20	−0.67	1.50
BLA and BNST	0.98	0.38	0.58	0.92
BLA and CeA	0.46	−0.21	−0.71	0.92
dCA1 and BNST	1.30	−0.12	−0.57	1.44
dCA1 and CeA	0.16	0.57	−0.29	1.02
dCA1 and BLA	1.07	0.35	1.91	−0.21
dCA2/3 and BNST	−0.31	1.90	−1.22	2.66
dCA2/3 and CeA	−0.21	−0.44	−0.81	0.00
dCA2/3 and BLA	−0.01	0.57	−0.30	0.87
dCA2/3 and dCA1	−0.42	1.82	0.82	0.78
dDG and BNST	0.09	2.30	−0.27	2.60
dDG and CeA	0.37	0.20	1.26	−0.48
dDG and BLA	−1.04	0.68	−1.35	0.89
dDG and dCA1	−0.23	1.67	0.69	0.90
dDG and dCA2/3	0.61	1.22	−0.13	1.84
PVN and BNST	−0.21	0.73	1.10	−0.53
PVN and CeA	−0.66	1.34	0.32	0.50
PVN and BLA	−1.23	0.98	−0.22	0.00
PVN and dCA1	−0.12	0.34	−2.61	2.43
PVN and dCA2/3	0.74	0.84	0.23	1.27
PVN and dDG	−0.35	0.64	−0.20	0.52
PVT and BNST	0.25	−1.82	0.54	−1.98
PVT and CeA	−0.73	−0.03	0.34	−1.13
PVT and BLA	−2.23	−0.39	−2.14	−0.80
PVT and dCA1	0.98	−0.88	0.80	−0.73
PVT and dCA2/3	−0.08	−0.80	1.00	−1.70
PVT and dDG	−0.83	−1.38	−0.45	−1.69
PVT and PVN	0.45	−1.19	−0.85	0.07
LS and BNST	−0.02	1.71	−0.47	2.05
LS and CeA	0.87	−0.25	0.36	0.33
LS and BLA	2.89	−0.11	1.34	1.77
LS and dCA1	−1.02	0.84	−1.78	1.52
LS and dCA2/3	0.15	0.71	−1.05	1.79
LS and dDG	0.90	1.38	0.82	1.44
LS and PVN	−1.27	1.94	1.48	−0.85
LS and PVT	1.44	−1.59	2.26	−2.39
MS and BNST	−0.83	−0.60	−0.49	−0.85
MS and CeA	0.17	0.63	−0.42	1.31
MS and BLA	2.53	−1.93	0.42	0.25
MS and dCA1	−0.58	0.57	−0.75	0.72
MS and dCA2/3	−0.29	0.19	−0.08	0.00
MS and dDG	0.91	−0.10	−0.41	1.07
MS and PVN	−0.70	2.47	0.86	0.80
MS and PVT	0.72	0.45	1.23	−0.14
MS and LS	−0.32	−0.88	−1.06	−0.07
MeA and BNST	−1.05	0.01	−0.78	−0.37
MeA and CeA	−1.15	−0.62	0.33	−2.22
MeA and BLA	0.11	0.68	1.40	−0.42
MeA and dCA1	−1.40	−0.73	−1.40	−0.96
MeA and dCA2/3	−0.76	−0.62	−0.88	−0.67
MeA and dDG	0.56	−0.61	2.19	−1.96
MeA and PVN	1.24	−2.10	0.13	−1.15
MeA and PVT	0.91	0.71	1.10	0.72
MeA and LS	−1.49	−0.21	−0.82	−1.07
MeA and MS	−1.42	−0.08	−2.03	0.35

## Results

### Spatial memory deficits provoked by multiple acute simultaneous stresses (MAS) differ across the estrous cycle

In order to test the potential role of the estrous cycle in the effects of MAS on spatial memory, female mice underwent MAS either on entering proestrus or during estrus, phases associated with high and low physiological levels of estradiol, respectively, and were then tested for spatial memory. In the OLM task (Fig. 1*A*), control mice at both cycle phases performed well. Mice experiencing MAS during early proestrus had poor spatial memory whereas those exposed to MAS during estrus were protected. We found an interaction of cycle phase × MAS on OLM performance (*F*_(1,35)_ = 5.78, *p* = 0.02) and no main effects of cycle phase (*F*_(1,35)_ = 2.24, *p* = 0.14) or MAS (*F*_(1,35)_ = 1.66, *p* = 0.21; Fig. 1*B*). *Post hoc* testing indicated a difference in performance between MAS and control in the early proestrus group (*t*_(35)_ = 2.58, *p* = 0.03) but not the estrus group (*t*_(35)_ = 0.80, *p* = 0.68). Furthermore, the early proestrus MAS group had significantly impaired performance compared with the estrus MAS group (*t*_(35)_ = 2.87, *p* = 0.01), while there were no differences in between control mice of both phases (*t*_(25)_ = 0.62, *p* = 0.79).

Notably, differences in OLM were not attributable to differences in exploration or object bias. During the training session, the ratio of time spent exploring the object moved during testing over the object that stayed in place did not differ among groups (estrus control 1.05 ± 0.04, proestrus control 1.09 ± 0.08, estrus MAS 1.03 ± 0.05, and proestrus MAS 0.99 ± 0.07), with no cycle × MAS interaction (*F*_(1,35)_ = 0.42, *p* = 0.52), main effect of cycle phase (*F*_(1,35)_ = 0.004, *p* = 0.95), or MAS (*F*_(1,35)_ = 0.79, *p* = 0.38). Similarly, total object exploration times during training (estrus control 21.51 ± 1.27 s, proestrus control 24.40 ± 2.54 s, estrus MAS 23.66 ± 1.21 s, proestrus MAS 20.65 ± 1.24 s) did not distinguish the groups, with no cycle × MAS interaction (*F*_(1,35)_ = 3.35, *p* = 0.08), main effect of cycle phase (*F*_(1,35)_ = 0.001, *p* = 0.97), or MAS (*F*_(1,35)_ = 0.24, *p* = 0.63). The testing phase was also not confounded by total object exploration times (estrus control 12.55 ± 0.62 s, proestrus control 16.32 ± 1.58 s, estrus MAS 15.10 ± 1.24 s, proestrus MAS 14.71 ± 1.60 s), with no cycle phase × MAS interaction (*F*_(1,35)_ = 2.41, *p* = 0.13), main effect of cycle phase (*F*_(1,35)_ = 1.60, *p* = 0.21), or MAS (*F*_(1,35)_ = 0.13, *p* = 0.72). Therefore, we concluded that MAS selectively impaired OLM of female mice during early proestrus but not during estrus.

The design of the OLM test involves training the females on the day of stress (early proestrus or estrus), and testing 24 h later, when cycle phase and associated estrogen levels might differ. Therefore, to determine with greater precision the contribution of specific cycle phases to the effects of MAS on memory, in a separate cohort of female mice we conducted a second, independent test of spatial memory in which both training and testing take place on the same day. During the testing phase of the spatial Y-maze task (Fig. 1*C*), 87.5% of control estrus mice and 75% of control early proestrus mice entered the novel arm first from the home arm of the apparatus. Of the MAS groups, 71.4% of estrus MAS mice versus 33.3% of early proestrus MAS mice entered the novel arm first (Fig. 1*D*). Both control groups and the estrus MAS group, but not the early proestrus MAS group entered the novel arm significantly more often than the familiar arm. A three-way ANOVA indicated a significant main effect of arm (*F*_(1,28)_ = 33.32, *p* < 0.0001; Fig. 1*E*). There were no interactions of arm × MAS × cycle phase (*F*_(1,28)_ = 3.20, *p* = 0.08), arm × MAS (*F*_(1,28)_ = 0.52, *p* = 0.48), MAS × cycle phase (*F*_(1,28)_ = 2.74, *p* = 0.11), or arm × cycle phase (*F*_(1,28)_ = 0.41, *p* = 0.53), and no main effects of cycle phase (*F*_(1,28)_ = 0.31, *p* = 0.58) or MAS (*F*_(1,28)_ = 2.64, *p* = 0.12). *Post hoc* testing indicated a significant preference for entries into the novel versus the familiar arm in both control groups: estrus (*t*_(28)_ = 2.69, *p* = 0.047) and early proestrus (*t*_(28)_ = 3.84, *p* = 0.003), as well as the estrus MAS group (*t*_(28)_ = 3.51, *p* = 0.006) but not in the proestrus MAS group (*t*_(28)_ = 1.40, *p* = 0.53). Thus, as found for the OLM, MAS selectively impaired spatial memory in early proestrous female mice in the Y-maze, while sparing mice in estrus.

These spatial Y-maze memory impairments were not attributable to differences in exploration of the apparatus or overall locomotion during the training or testing sessions of the task. Total entries into the open and home arms during training were equivalent: estrus control 70.63 ± 11.54, proestrus control 79.13 ± 14.44, estrus MAS 87.71 ± 6.86, and proestrus MAS 93.00 ± 7.03, with no cycle phase × MAS interaction (*F*_(1,28)_ = 0.02, *p* = 0.88), main effect of cycle phase (*F*_(1,28)_ = 0.43, *p* = 0.52), or MAS (*F*_(1,28)_ = 2.16, *p* = 0.15). Distance traveled during the training phase was equivalent across all groups (estrus control 3500 ± 250.0 cm, proestrus control 3395 ± 235.7 cm, estrus MAS 3435 ± 179.8 cm, and proestrus MAS 3807 ± 177.0 cm), with no cycle phase × MAS interaction (*F*_(1,28)_ = 1.24, *p* = 0.28), effect of MAS (*F*_(1,28)_ = 0.65, *p* = 0.43), or of cycle phase (*F*_(1,28)_ = 0.39, *p* = 0.54). During the testing session, there was an effect of MAS on total entries (into the novel, familiar, and home arms: estrus control 34.38 ± 4.76, proestrus control 40.25 ± 5.33, estrus MAS 49.71 ± 3.79, and proestrus MAS 43.22 ± 1.77, *F*_(1,28)_ = 5.05, *p* = 0.03), but no cycle phase × MAS interaction (*F*_(1,28)_ = 2.30, *p* = 0.14) or effect of cycle phase (*F*_(1,28)_ = 0.006, *p* = 0.94). The effect of MAS was significant in the estrus group (*t*_(28)_ = 2.58, *p* = 0.03) but not in proestrus (*t*_(28)_ = 0.53, *p* = 0.84). However, the total number of entries did not differ between the estrus MAS and the proestrus MAS groups (*t*_(28)_ = 1.12, *p* = 0.47) and is thus unlikely to explain the discrepancy in memory performance between these two groups. During the testing session, there were no differences in distance traveled (estrus control 2014 ± 181.8 cm, proestrus control 2235 ± 269.5 cm, estrus MAS 2358 ± 92.91 cm, and proestrus MAS 2165 ± 75.48 cm) with no cycle phase × MAS interaction (*F*_(1,28)_ = 2.51, *p* = 0.12), effects of cycle phase (*F*_(1,28)_ = 0.12, *p* = 0.73), or MAS (*F*_(1,28)_ = 0.29, *p* = 0.59). Together, these data dismiss the likelihood that reduced exploration explains the impaired performance in the Y-maze of early proestrous mice subjected to MAS.

Together, the results of the two independent measures of spatial memory demonstrated impaired spatial memory in mice exposed to MAS during early proestrus but not during estrus.

### Physiologic estradiol levels are high in early-proestrous mice that have impaired memory following MAS

The results above, in which mice in a cycle phase when estrogen levels are high (early proestrus) had impaired spatial memory following MAS, were unexpected. Indeed, we chose to test female mice in these two cycle phases with the expectation that higher estrogen levels might protect memory in female mice from the impact of MAS, given that estrogen has been shown to enhance memory processes and the structure and function of hippocampal neurons (Vierk et al., 2014; Wang et al., 2018) and protect against stress-induced memory impairments (Wei et al., 2014). Female mice in estrus, however, had no MAS-induced memory disturbances. To verify the congruence of our categorization and estrogen levels, we measured serum estradiol in independent cohorts of mice which were carefully classified for cycle phase based on daily vaginal smears for a minimum of two cycles. To further establish the cyclic physiological functions of estradiol, we harvested uteri and determined the estrogen-dependent uterine weight and uterine index.

**Figure 2.**
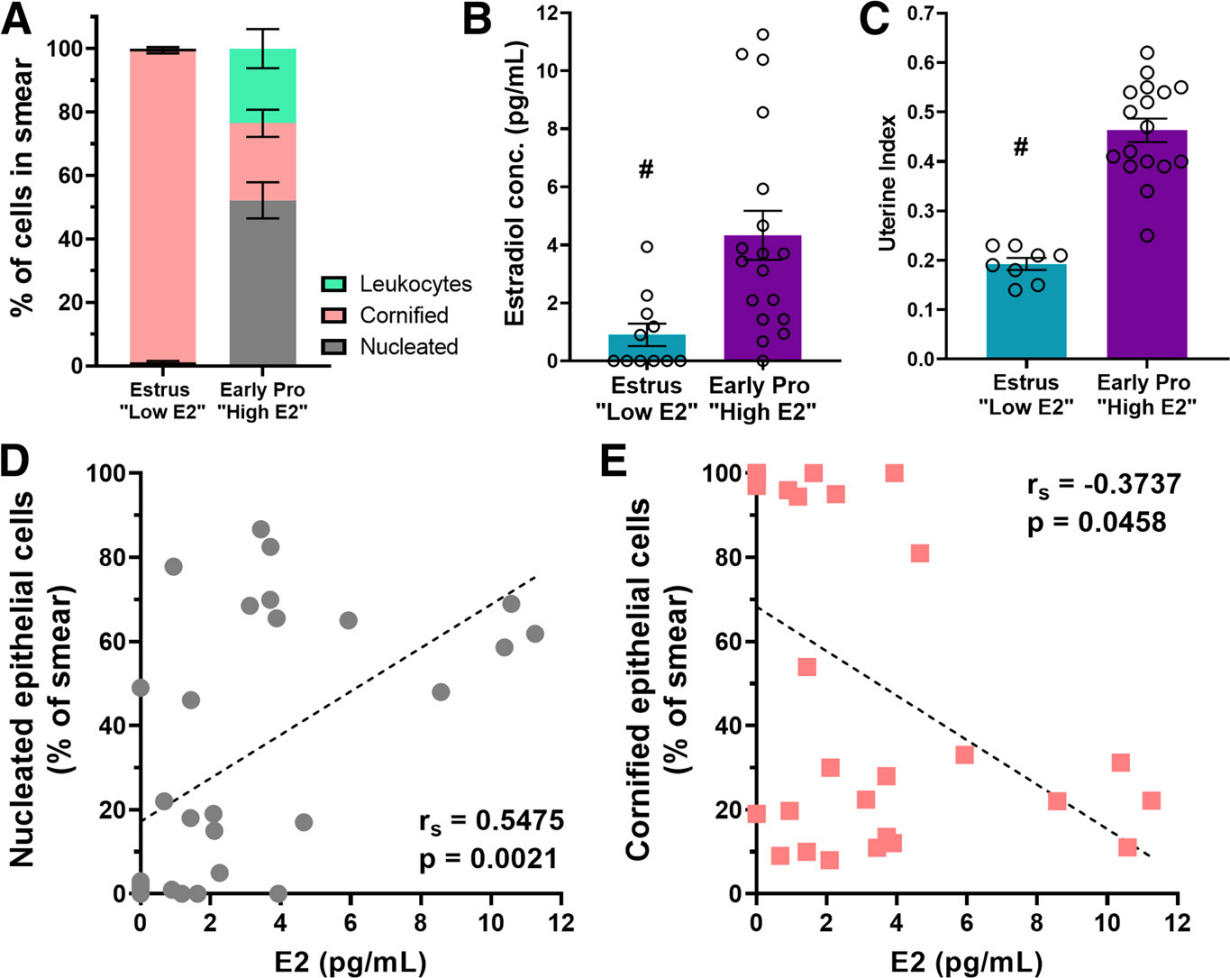
Early proestrous mice (those with impaired memory following MAS) have higher levels of circulating estradiol. Estrous cycles were monitored via vaginal cytology and at the time of the experiment mice were divided into groups that were early proestrus or estrus. ***A***, Vaginal cytology classifications were done according to relative presence of nucleated epithelial, cornified epithelial, or leukocytes in the sample. High E2 mice had a majority nucleated cells, whereas low E2 mice had smears consisting of almost entirely cornified cells (*n* = 11-18/group). ***B***, Mice classified as early proestrus according to their vaginal smears had higher average estradiol in serum samples as measured by an estradiol ELISA compared with estrus mice (*n* = 11-18 mice/group). ***C***, High E2 mice had higher average uterine indices (uterus weight/body weight × 100) (*n* = 8-17 mice per group). ***D***, The amount of estradiol within a sample had a significant positive correlation with the percentage of the smear that consisted of nucleated epithelial cells. ***E***, Estradiol had a negative correlation with cornified cells. From this point on, early proestrus mice were classified as high E2 and estrus mice were classified as low E2. ^#^*p* < 0.05, main effect. Points represent scores of individual animals. Error bars indicate ± SEM.

First, we established consistent and rigorous cycle phases by quantifying the cell type composition in vaginal smears. Estrous phase smears consisted predominantly of cornified cells. Early proestrous phase smears had a large proportion of nucleated cells with some leukocytes and some cornified cells. There was an interaction of cycle phase × cell type (*F*_(2,54)_ = 57.91, *p* < 0.0001) and an effect of cell type (*F*_(1.737,46.91)_ = 34.26, *p* < 0.0001), but no effect of cycle (*F*_(1,27)_ = 0.5237, *p* = 0.48; Fig. 2*A*). *Post hoc* tests indicated that the numbers of nucleated (*t*_(17.25)_ = 8.92, *p* < 0.0001), cornified cells (*t*_(17.75)_ = 16.98, *p* < 0.0001), and leukocytes (*t*_(17.28)_ = 3.69, *p* = 0.005) differed between estrous and early proestrous mice. Because stress can alter the duration of the estrous cycle (Breen et al., 2012), we assessed this parameter in MAS mice: we monitored each female for 3 or 4 cycles before and after MAS in a preliminary experiment. The average cycle length did not change from before to after MAS (pre: 5.19 ± 0.07 d, post: 5.23 ± 0.23 d) and did not differ from a control group (pre: 5.00 ± 0.13 d, post: 4.81 ± 0.17 d). Time point × MAS interaction (*F*_(1,27)_ = 0.53, *p* = 0.47), effect of time point (*F*_(1,27)_ = 0.27, *p* = 0.61), and effect of MAS (*F*_(1,27)_ = 2.87, *p* = 0.10) were not statistically significant. Therefore, we concluded that MAS did not alter the length of the estrous cycle.

We then measured serum estradiol in regularly cycling female mice. Early proestrous mice had higher concentrations of serum estradiol than those in estrus (*U* = 35, *p* = 0.002; Fig. 2*B*). Further, serum estradiol levels correlated with the vaginal smear cell type composition across both phases of the estrous cycle: estradiol levels of an individual mouse were positively correlated with the percentage of nucleated epithelial cells in vaginal smears from the same mouse (Spearman: *r*_s_ = 0.55, *p* = 0.002, Pearson: *R*^2^ = 0.30, *p* = 0.002; Fig. 2*D*) and negatively correlated with percentage of cornified cells (Spearman: *r*_s_ = −0.37, *p* = 0.046, Pearson: *R*^2^ = 0.21, *p* = 0.014; Fig. 2*E*). Seeking a second, independent biological marker of estradiol levels, we determined uterine weights, which have been shown to fluctuate across the estrous cycle and depend on systemic estrogen levels (Balmain et al., 1956; Galloa et al., 1986; Lemini et al., 2015). Uterine indices (uterus weight (g)/body weight (g) × 100) of mice entering proestrus were greater than those in estrus (*t*_(23)_ = 7.52, *p* < 0.0001; Fig. 2*C*). Thus, using vaginal cytology matched with serum estradiol or uterine weights, we categorized female mice in estrus as low estradiol (E2) and mice in early proestrus as high estradiol (E2).

### MAS-provoked loss of hippocampal dendritic spines aligns with spatial memory impairment

In male mice, in which hippocampal estrogen levels are higher than in proestrous females (Hojo et al., 2004; Kato et al., 2013), MAS-induced spatial memory deficits strongly correlate with loss of apical dendritic spines in dorsal hippocampal CA1 fields. In addition, the spine loss is most prominent for thin spines, considered to undergo plasticity during memory acquisition (Bourne and Harris, 2007; Kasai et al., 2010; Maras et al., 2014; Chen et al., 2016). Spine density is thought to be a proxy for the density of excitatory synapses. Therefore, we tested the effects of MAS on apical dendritic spine densities in low E2 and high E2 female mice.

**Figure 3.**
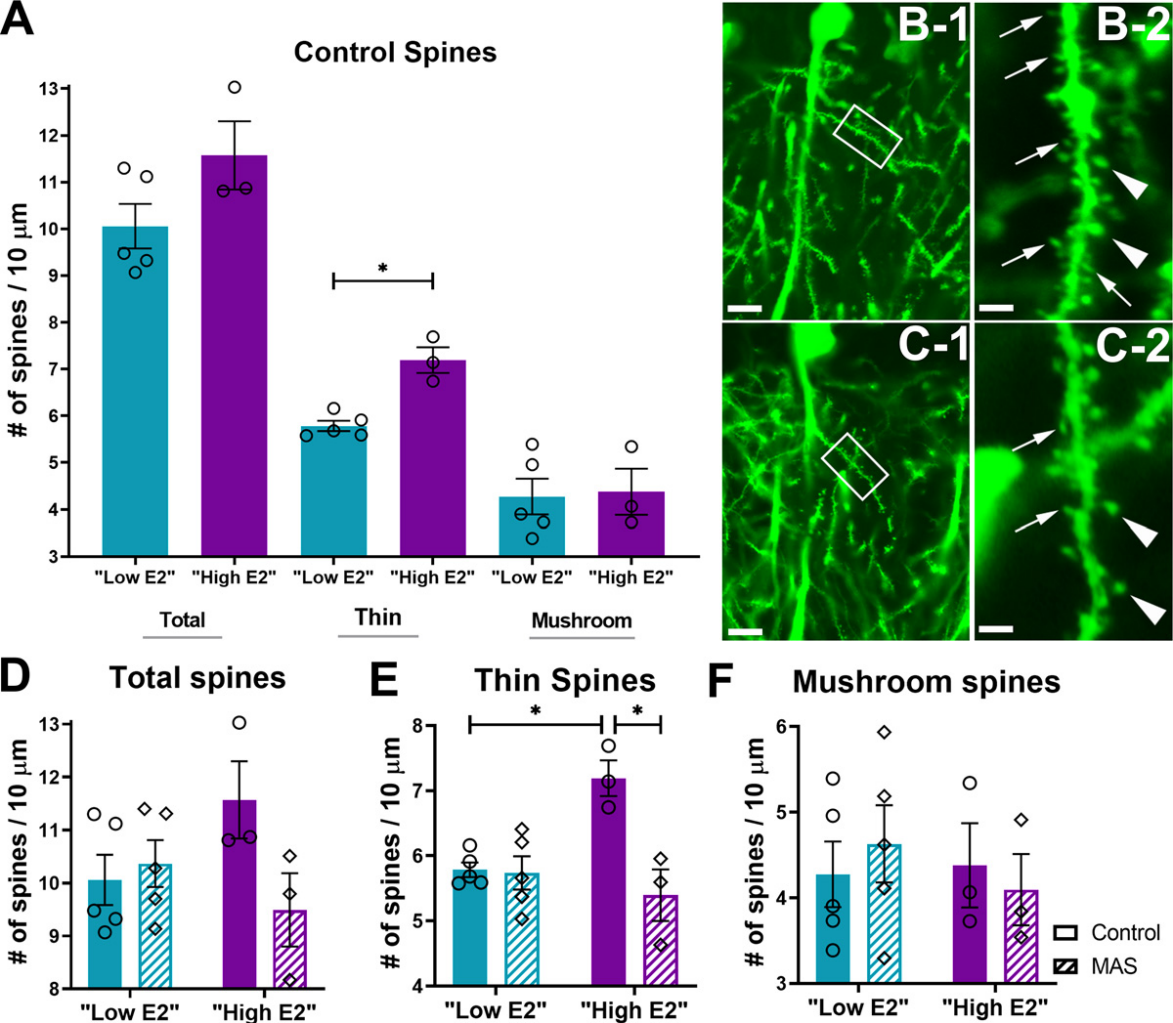
Dendritic spine loss in CA1 of the dorsal hippocampus is induced by MAS in high E2 but not low E2 female mice. Dendritic spines were visualized in mice expressing YFP in pyramidal cells under the control of the Thy1 promoter. ***A***, Under control conditions, mice with higher levels of estrogen had more thin spines but no difference in mushroom or total (thin + mushroom) spines. ***B***, High E2 control mice had thin (thin arrows) and mushroom spines (thick arrows) in the stratum radiatum of CA1. ***C***, After MAS, high E2 mice selectively lost thin spines. Framed areas are enlarged in ***B-2*** and ***C-2***. Scale bars: ***B-1***, ***C-1***, 10 µm; ***B-2***, ***C-2***, 2 µm. ***D***, Following 2 h MAS, there was no significant difference in total number of spines between control or MAS mice in either cycle phase. ***E***, Thin spines were greater in high E2 control mice than low E2 control mice, but these spines were selectively reduced following MAS in the high E2 phase. ***F***, Mushroom spines remained intact following MAS in either cycle phase (*n* = 3-5 mice/group). *p < 0.05 (post-test). Points represent scores of individual animals. Error bars indicate ± SEM.

First, we examined whether estrous cycle phases themselves influenced dendritic spine density in female mice. In rats, hippocampal dendritic spine density has been found to fluctuate across the estrous cycle peaking during proestrus (Gould et al., 1990; Woolley et al., 1990). Therefore, we compared spine densities with regards to estrous cycle phase in unstressed female mice using planned comparison post-tests for the ANOVAs of each spine subtype. Mean densities of total or mushroom spines did not differ between low E2 and high E2 control mice (total: *t*_(12)_ = 1.882, *p* = 0.16; mushroom: *t*_(12)_ = 0.16, *p* = 0.98). However, densities of thin spines were significantly higher in high E2 versus low E2 mice (*t*_(12)_ = 3.88, *p* = 0.004; Fig. 3*A*). We then determined the effects of MAS on the same dendritic spine subtypes in low and high E2 female mice.

MAS reduced spine densities in apical dendrites from dorsal CA1 of high E2 mice. Whereas cycle phase × MAS interaction (*F*_(1,12)_ = 4.41, *p* = 0.058) and main effects of cycle phase (*F*_(1,12)_ = 0.32, *p* = 0.58) and of MAS (*F*_(1,12)_ = 2.43, *p* = 0.15; Fig. 3*D*) were not significantly different for total spine counts, thin spines were significantly affected. For thin spines, a significant cycle phase × MAS interaction was identified (*F*_(1,12)_ = 11.65, *p* = 0.005) as well as a main effect of MAS (*F*_(1,12)_ = 12.99, *p* = 0.004), but not for cycle phase (*F*_(1,12)_ = 4.32, *p* = 0.06). The difference in thin spine densities between the control and MAS groups was confined to the high E2 mice (*t*_(12)_ = 4.44, *p* = 0.002; Fig. 3*B*,*C*), and not observed in low E2 mice (*t*_(12)_ = 0.16, *p* > 0.99; Fig. 3*E*). MAS reduced thin spines in high E2 mice compared with control density; however, thin spine density did not differ between low E2 MAS and high E2 MAS mice (*t*_(12)_ = 0.94, *p* = 0.94). Mushroom spines were unaffected (cycle × MAS interaction: *F*_(1,12)_ = 0.48, *p* = 0.50; cycle: *F*_(1,12)_ = 0.22, *p* = 0.65; or MAS: *F*_(1,12)_ = 0.006, *p* = 0.94; Fig. 3*F*). Thus, in accord with the findings in male mice, in which hippocampal estradiol is high and MAS impair memory, thin dendritic spines were reduced after MAS only in high E2 female mice in which memory was compromised by MAS.

### Differential hippocampal activation during MAS does not explain estrous cycle-dependent memory impairment

The estrous cycle has been shown to influence responses to stress in female rodents (Heck and Handa, 2019). As both spatial memory deficits and thin spine loss provoked by MAS involved the dorsal hippocampus, we tested whether MAS led to augmented activation of hippocampal neurons in high E2 compared with low E2 female mice.

**Figure 4.**
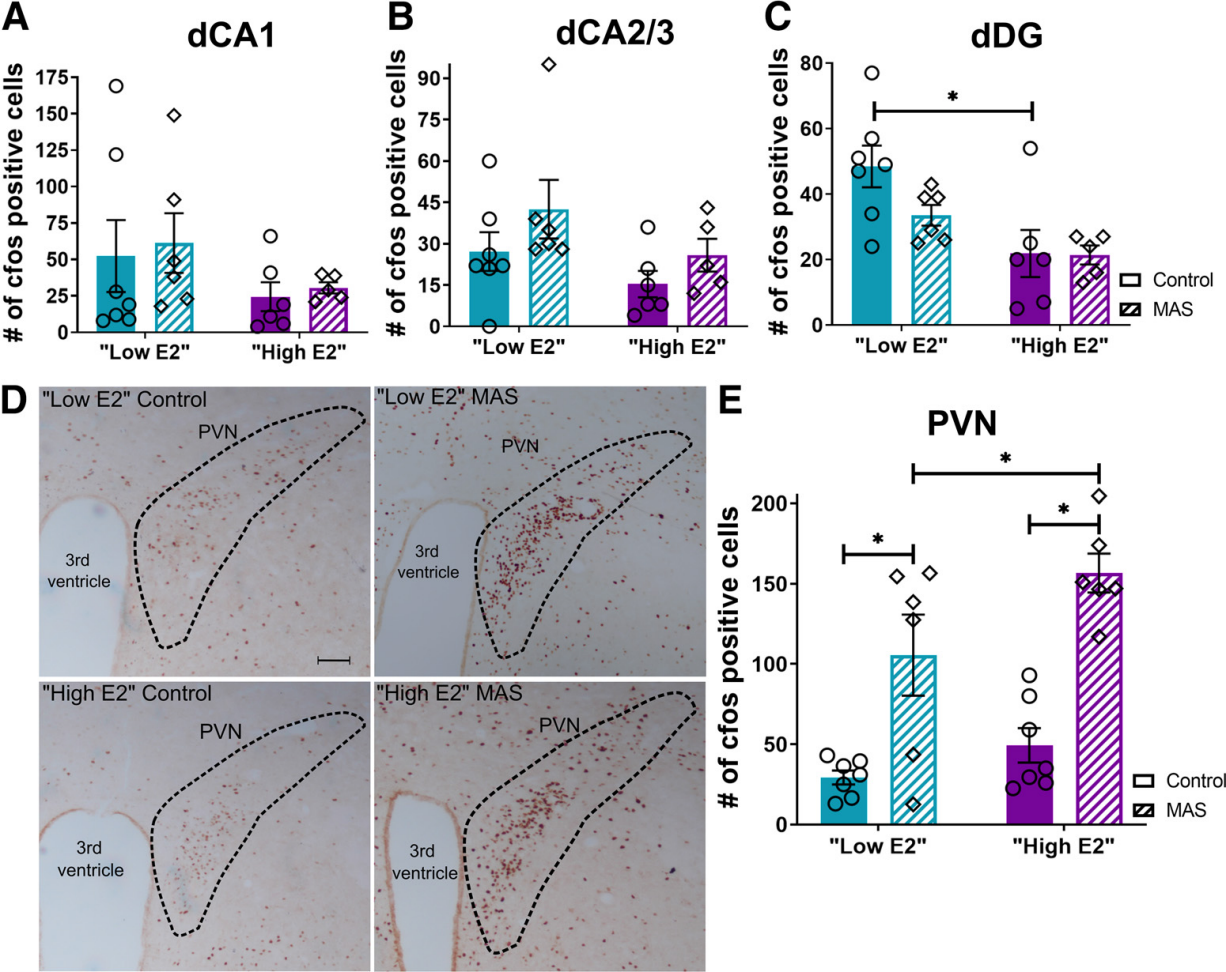
MAS-induced memory impairments are not explained by differential activation of the dorsal hippocampus. Activation of the dorsal hippocampus and hypothalamic paraventricular nucleus (PVN) was assessed by quantifying *fos*^+^ cells in control or MAS mice in either cycle phase. ***A***, *fos*^+^ cells did not differ with cycle phase or MAS in the CA1 region. ***B***, Numbers of *fos*^+^ cells also did not differ with cycle phase or MAS in the CA2 and CA3 regions. ***C***, In the DG, there was an effect of cycle on *fos*^+^ cells, with cell number distinguishing low E2 and high E2 in control but not MAS mice. ***D***, ***E***, *fos*^+^ cells in the hypothalamic PVN were more abundant following MAS in both groups, with a greater increase in activation in the high E2 group (*n* = 5-7 mice per group). Scale bar, 100 µm. *p < 0.05 (post-test). Points represent scores of individual animals. Error bars indicate ± SEM.

Neuronal activation immediately following MAS was assessed using levels of the activity-regulated gene product *fos* as in index of neuronal activity. The effects of MAS and estrous cycle phases on *fos* expression were examined across subregions of the dorsal hippocampus. In CA1, a field critical for spatial memory and the site of spine loss following MAS in high E2 female mice, there were no effects of MAS, cycle, or any interactions (cycle × MAS interaction: *F*_(1,20)_ = 0.006, *p* = 0.94; cycle: *F*_(1,20)_ = 2.45, *p* = 0.13; MAS: *F*_(1,20)_ = 0.16, *p* = 0.69; Fig. 4*A*). Similarly, the number of *fos*^+^ cells in combined CA2 and CA3 did not differ in regards to cycle phase or MAS (cycle × MAS interaction: *F*_(1,20)_ = 0.10, *p* = 0.75; cycle: *F*_(1,20)_ = 3.51, *p* = 0.08; MAS: *F*_(1,20)_ = 2.88, *p* = 0.11; Fig. 4*B*). Finally, in the DG, there were more *fos*^+^ cells in low E2 mice than high E2 mice at baseline, with an effect of cycle (*F*_(1,20)_ = 11.88, *p* = 0.003), but no cycle × MAS interaction (*F*_(1,20)_ = 1.67, *p* = 0.21) and no effect of MAS (*F*_(1,20)_ = 1.87, *p* = 0.19; Fig. 4*C*). *Fos* cell counts differed in the DG between low high E2 mice in control (*t*_(20)_ = 3.50, *p* = 0.005) but not MAS (*t*_(20)_ = 1.46, *p* = 0.29) mice.

To determine whether the MAS protocol used here indeed led to significant neuronal activation measurable by *fos* expression levels, we quantified MAS-induced *fos*^+^ cells in the hypothalamic PVN, an established stress-responsive brain region. The number of *fos*^+^ cells in PVN of MAS experiencing mice was significantly higher than that in control mice (Fig. 4*D*). The number of *fos*^+^ cells in the PVN demonstrated main effects of cycle (*F*_(1,22)_ = 6.21, *p* = 0.02) and of MAS (*F*_(1,22)_ = 41.26, *p* < 0.0001; Fig. 4*E*), without cycle × MAS interaction (*F*_(1,22)_ = 1.18, *p* = 0.29). Specifically, the number of *fos*^+^ cells was higher in MAS versus control mice in both cycle phases (low E2: *t*_(22)_ = 3.77, *p* = 0.002; high E2: *t*_(22)_ = 5.31, *p* < 0.0001). Additionally, the number of *fos*^+^ cells following MAS was higher in high E2 compared with low E2 mice (*t*_(22)_ = 2.44, *p* = 0.046). This cycle difference was not observed in unstressed controls (*t*_(22)_ = 1.03, *p* = 0.53). Thus, these data demonstrated that MAS leads to neuronal activation within salient brain regions; and, for the PVN but not for the hippocampus, high E2 mice have enhanced MAS-driven neuronal activation.

Importantly, these analyses demonstrated that, although hippocampal memory impairment and dendritic spine loss were observed preferentially in high E2 mice experiencing MAS, these effects were not a result of differential hippocampal activation patterns.

### Estrous cycle phase influences neuronal activation in salient brain regions, and modulates stress-induced activation

Stress-induced dendritic spine loss requires glutamate receptor-mediated neuronal activation (Andres et al., 2013). Given that hippocampal activation did not explain the behavioral memory impairments and dendritic spine loss observed, we examined activation in salient brain regions, defined as those afferent to, or interconnected with, the hippocampus that might be differentially affected by MAS or estrous cycle phase and thus drive functional hippocampal impairment. We chose *a priori* brain regions involved in stress and memory which are interconnected with the hippocampus and determined *fos* expression in several regions from the same mouse. We identified a significant brain region × MAS × cycle interaction (*F*_(10 206)_ = 3.071, *p* = 0.001) in a three-way ANOVA, and therefore analyzed each region independently.

**Figure 5.**
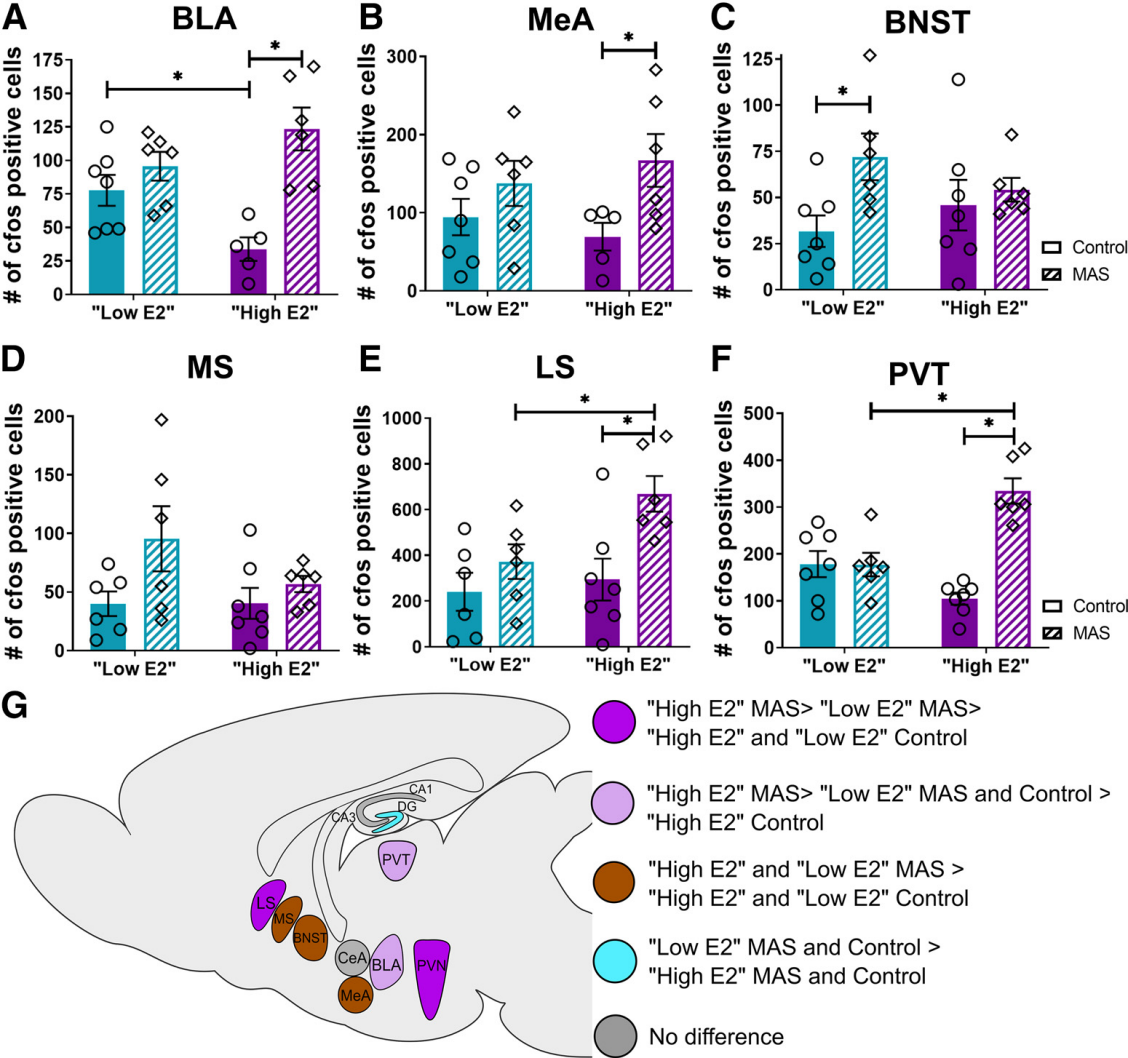
Neuronal activation across the brain varies with cycle phase and in response to MAS. *fos*^+^ cells were quantified in the BLA, MeA, BNST, MS, LS, and the PVT. ***A***, Whereas there were fewer *fos*^+^ cells in BLA at baseline in the high E2 group, MAS resulted in a significant increase in *fos*^+^ cells in this group only. ***B***, In the MeA, MAS increased the number of *fos*^+^ cells significantly in high E2 mice but not in low E2 mice. ***C***, In the BNST, MAS increased *fos*^+^ cell numbers in the low E2 group only. ***D***, In the MS, there was a main effect of MAS that did not differ between mice at different cycle phases. ***E***, In the LS, there was an increase in *fos*^+^ cells following MAS in the high E2 group, but not the low E2 group. ***F***, In the PVT, the number of *fos*^+^ cells was augmented by MAS in the high E2 group only. ***G***, Graphic summary of differences in *fos* counts across brain regions (*n* = 5-7 mice per group). *p < 0.05 (post-test). Points represent scores of individual animals. Error bars indicate ± SEM.

The amygdala is a key node of emotional processing and is highly susceptible to stress (Zhang et al., 2018). *fos*^+^ cells were quantified in select nuclei of the amygdala and the extended amygdala in control and MAS-experiencing mice at both high and low E2 cycle phases. The CeA plays a key role in stress responses. Analyzing the number of *fos*^+^ cells in this nucleus, we identified a significant cycle × MAS interaction (*F*_(1,20)_ = 4.48, *p* = 0.047), but no effect of cycle (*F*_(1,20)_ = 0.81, *p* = 0.38) or MAS (*F*_(1,20)_ = 0.01, *p* = 0.91). Despite the significant interaction of MAS and cycle, *fos* cell number did not differ in the CeA between control and MAS in either cycle phase (high E2: *t*_(20)_ = 1.52, *p* = 0.27; low E2: *t*_(20)_ = 1.48, *p* = 0.29). The number of *fos*^+^ cells for the CeA were as follows: low E2 control 26.71 ± 5.38, high E2 control 20.80 ± 5.85, low E2 MAS 17.00 ± 2.77, and high E2 MAS 31.67 ± 4.71. We then analyzed *fos* expression as a function of cycle phase and MAS in the BLA, which projects robustly to both ventral and dorsal hippocampus (Pikkarainen et al., 1999; Petrovich et al., 2001; Beyeler et al., 2018). In the BLA, there was a significant cycle phase × MAS interaction (*F*_(1,20)_ = 8.34, *p* = 0.009), a main effect of MAS (*F*_(1,20)_ = 18.78, *p* = 0.0003), but no effect of cycle (*F*_(1,20)_ = 0.42, *p* = 0.52; Fig. 5*A*), on the number of *fos*^+^ cells. The numbers of *fos*^+^ cells were significantly increased by MAS in the BLA of high E2 female mice (*t*_(20)_ = 4.90, *p* = 0.0002), but not in low E2 mice (*t*_(20)_ = 1.07, *p* = 0.51). Notably, the number of *fos*^+^ cells was higher in control low E2 than high E2 mice (*t*_(20)_ = 2.48, *p* = 0.04). *fos*^+^ cell number in the BLA was not different between high E2 MAS and low E2 MAS mice (*t*_(20)_ = 1.60, *p* = 0.24).

In the MeA, there was a significant main effect of MAS (*F*_(1,20)_ = 6.65, *p* = 0.02) but no cycle × MAS interaction (*F*_(1,20)_ = 1.00, *p* = 0.33), or effect of cycle (*F*_(1,20)_ = 0.007, *p* = 0.94; Fig. 5*B*). *Fos* cells were significantly increased by MAS in the MeA of high E2 mice (*t*_(20)_ = 2.43, *p* = 0.049) but not in low E2 mice (*t*_(20)_ = 1.17, *p* = 0.45). The anterior division of the BNST, a component of the extended amygdala, may play a role in inhibiting the neuroendocrine stress response by inhibiting the PVN (Radley et al., 2009; Radley and Sawchenko, 2011). In the anterior BNST, there was an effect of MAS (*F*_(1,22)_ = 4.95, *p* = 0.04) but no cycle × MAS interaction (*F*_(1,22)_ = 2.14, *p* = 0.16), or effect of cycle (*F*_(1,22)_ = 0.03, *p* = 0.87; Fig. 5*C*). MAS increased *fos* cell number in the low E2 group (*t*_(22)_ = 2.61, *p* = 0.03) but not the high E2 group (*t*_(22)_ = 0.54, *p* = 0.84).

Septal nuclei, especially the medial septum (MS), are involved in the generation of the theta rhythm of the hippocampus, which supports memory processing (Courtin et al., 2014). The MS has bidirectional connectivity with the hippocampus, whereas the lateral septum (LS) only receives unidirectional afferents from the hippocampus, but the two septal subregions are interconnected (Tsanov, 2018; Agostinelli et al., 2019). The number of *Fos* cells in the MS was influenced by MAS (*F*_(1,21)_ = 4.82, *p* = 0.04), without cycle × MAS interaction (*F*_(1,21)_ = 1.41, *p* = 0.25) or effect of cycle (*F*_(1,21)_ = 1.37, *p* = 0.26; Fig. 5*D*). Despite an effect of MAS on *fos* in the MS, there were no significant differences following MAS in mice from either cycle phase (high E2: *t*_(21)_ = 0.73, *p* = 0.72; low E2: *t*_(21)_ = 2.35, *p* = 0.057). For *fos* cells in the LS, there was an effect of cycle (*F*_(1,21)_ = 4.37, *p* = 0.049) and of MAS (*F*_(1,21)_ = 9.18, *p* = 0.006) but no cycle × MAS interaction (*F*_(1,21)_ = 2.11, *p* = 0.16; Fig. 5*E*). The increase in *fos*^+^ cells following MAS was significant in the high E2 group (*t*_(21)_ = 3.23, *p* = 0.008) but not the low E2 group (*t_(_*_21)_ = 1.10, *p* = 0.49). The number of *fos*^+^ cells was higher in high E2 MAS mice than in low E2 MAS mice (*t*_(21)_ = 2.46, *p* = 0.04). Notably, there was no difference between controls at either cycle phase (*t*_(21)_ = 0.46, *p* = 0.88).

Arousing conditions including stress can activate the PVT, which is interconnected with the hippocampus (Kirouac, 2015). The number of *fos*^+^ cells in the PVT was significantly affected by MAS (*F*_(1,22)_ = 23.05, *p* < 0.0001) with no cycle × MAS interaction (*F*_(1,22)_ = 23.50, *p* < 0.0001), and no effect of cycle (*F*_(1,22)_ = 3.06, *p* = 0.09; Fig. 5*F*). The MAS-induced increase in *fos* was significant in the high E2 group (*t*_(22)_ = 6.82, *p* < 0.0001) but not the low E2 group (*t*_(22)_ = 0.03, *p* = 0.999). There were more *fos*^+^ cells in high E2 MAS than low E2 MAS mice (*t*_(22)_ = 4.49, *p* = 0.0004), but there was no difference between the control groups of either phase (*t*_(22)_ = 2.28, *p* = 0.06).

In summary, regions comprising nodes of the hippocampal network and those involved in stress processing responded to MAS in a region-specific and estrous cycle-dependent manner (data summarized graphically in Fig. 5*G*). Because the combinatorial activity of these regions and their projections to the hippocampus might predict or contribute to MAS-related loss of dendritic spines and spatial memory, we examined the functional connectivity of these regions, and determined functional network changes across cycle phases and as a result of MAS.

### Differential effects of cycle phase and MAS on functional networks among brain regions involved in stress and memory

**Figure 6.**
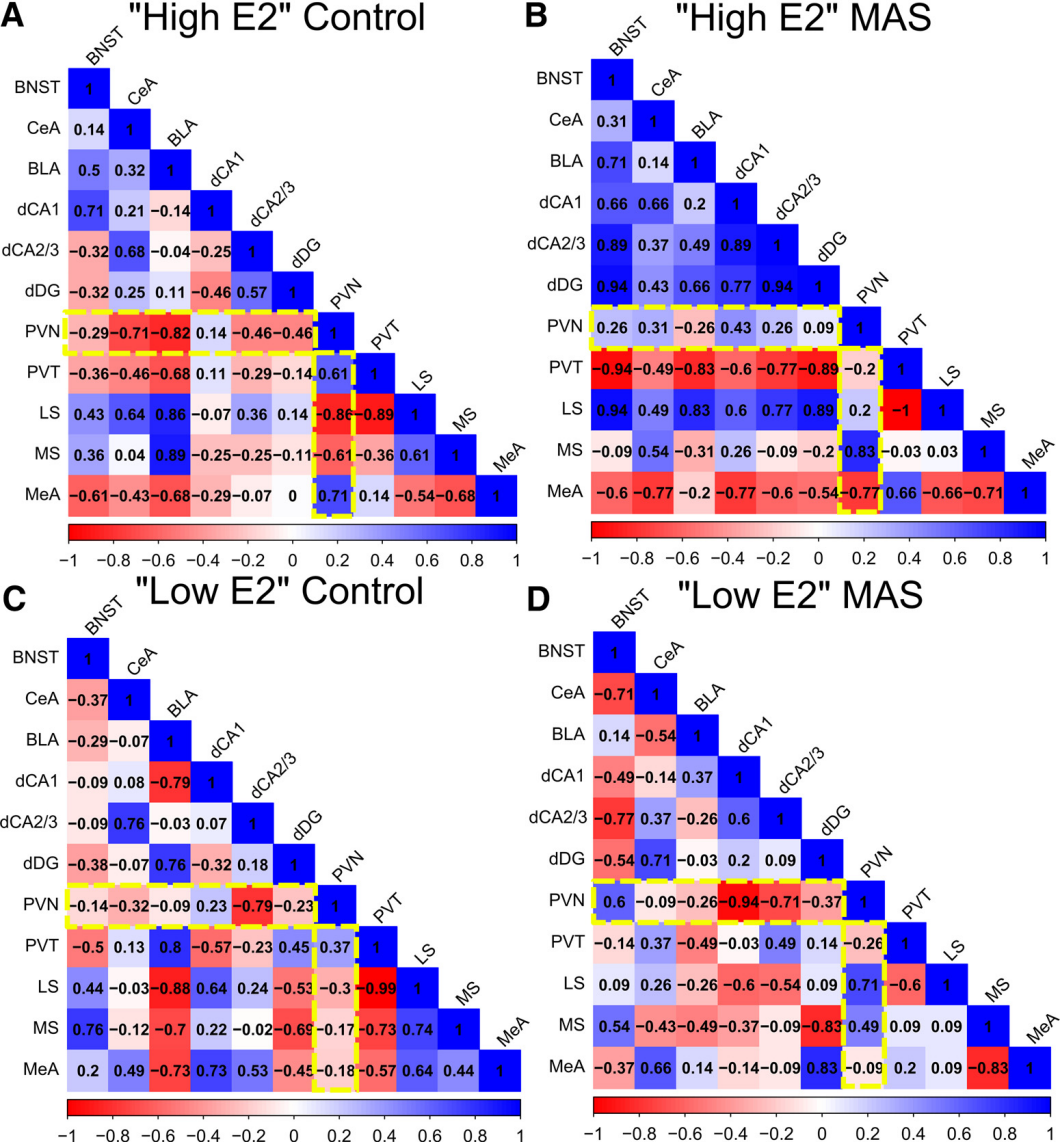
Correlated neuronal activity is influenced by estrous cycle phases and MAS. Patterns of neuronal activity were inferred by computing the Spearman correlations of scaled counts of *fos*^+^ cells among all brain regions. Within conditions, correlation matrices were computed for the following: ***A***, High E2 control. ***B***, High E2 MAS mice. ***C***, Low E2 control. ***D***, Low E2 MAS. As an example of MAS-induced changes of correlated activity, the yellow rectangles represents correlations with the PVN. Many PVN correlations are negative in high E2 control but shift to positive with MAS, whereas such a change is not evident among low E2 conditions.

Complex behaviors, including learning and memory, result from functional brain networks (Pattwell et al., 2016). Therefore, using *fos* expression as a marker of neuronal activity, we examined the presence of functional connections among the brain regions identified above. We used Spearman correlation matrices of *fos*^+^ cell numbers across all measured regions to identify coactivation between two given regions. Spearman correlation coefficients close to 1 indicated a positive relationship of activation between the two regions whereas values near −1 identified negative (anticorrelated) relations (Maras et al., 2014; Wiersielis et al., 2016; Salvatore et al., 2018; Ruiz et al., 2020). Correlation matrices were computed for each condition (high E2 control, high E2 MAS, low E2 control, and low E2 MAS; Fig. 6*A–D*). These correlation matrices provided evidence of coactivated regions associated with each individual condition. Importantly, they provided a method to compare the direction and strength of interregional coactivation as a function of MAS and estrous cycle phase. For example, correlations with the PVN (Fig. 6, yellow rectangle) are negative in high E2 control but shift to positive with MAS, whereas such a change is not evident among low E2 conditions.

We next examined the influence of cycle phase/estrogen levels and MAS on these putative functional networks. To compare two networks, correlation coefficients were converted to *z* scores (Fisher's *r* to *z* transformation), and the difference between these scores were calculated. A positive difference between the two *z* scores (indicating that the first group had a stronger functional relationship between the two regions than the second), is denoted in blue. A negative difference (the first group had a weaker relation), is denoted in red. In the absence of a difference, no connection is displayed. All differences in correlation coefficients (*Z*_obs_) are detailed in Table 1, and notable differences are elaborated on below.

**Figure 7.**
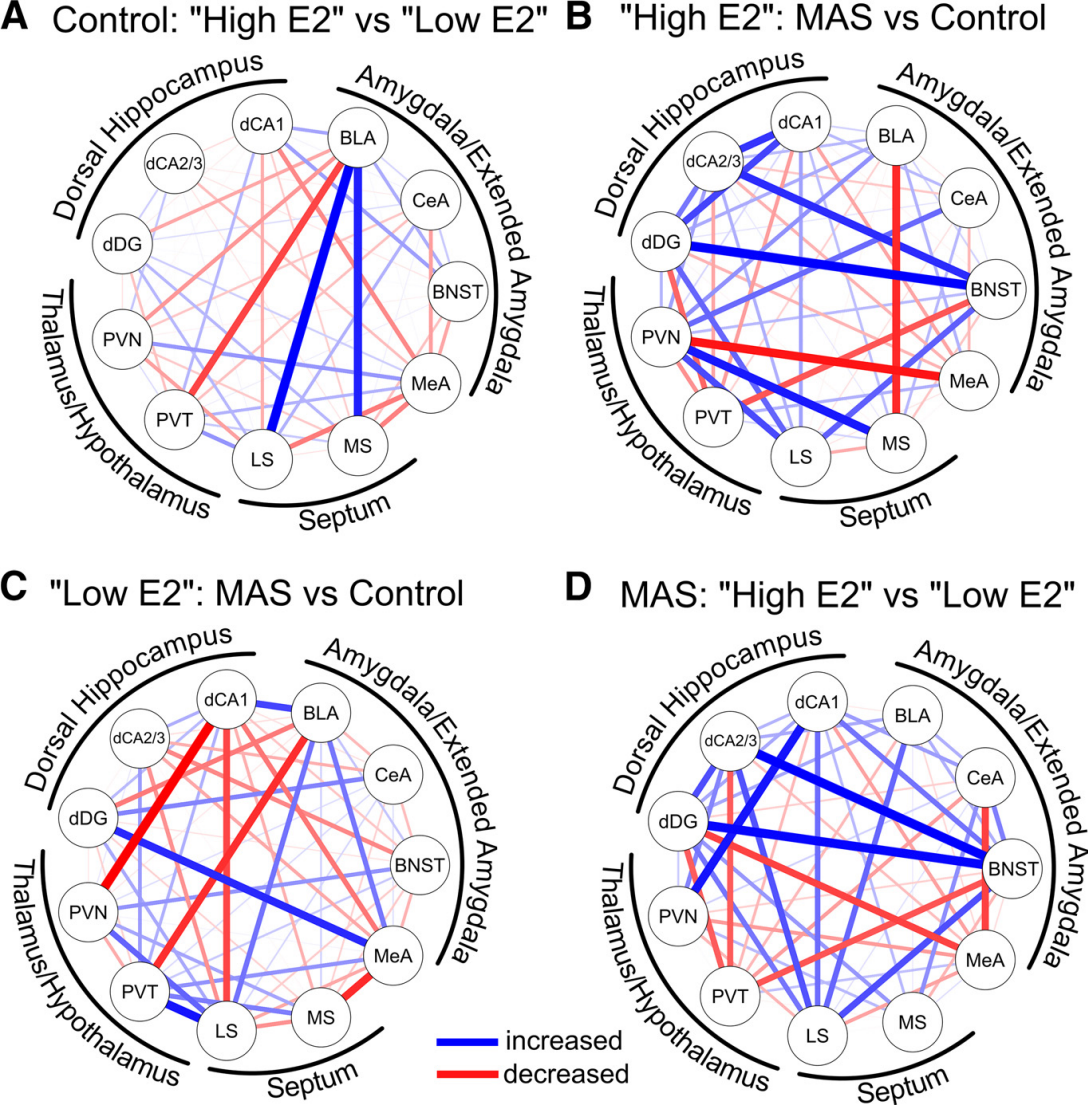
Comparing correlated neuronal activity among groups uncovers connectivity patterns that may contribute to MAS-induced memory impairments. To compare the differently active networks between conditions, Spearman correlations (see Fig. 6) were transformed to *z* scores that were compared between pairs of groups. Differential connectivity networks were constructed that indicated relationships that were increased (blue) or decreased (red) in Group 1 (first group listed) compared with Group 2 (second group listed). Line thickness indicates the intensity of this difference. Comparative functional networks were constructed for the following: ***A***, Control: high E2 compared with low E2. ***B***, High E2: MAS compared with control. ***C***, Low E2: MAS compared with control. ***D***, MAS: high E2 compared with low E2 mice.

Comparing control mice at high E2 versus low E2 cycle phases (Fig. 7*A*), we identified the BLA as a strongly connected hub in high E2 mice. Specifically, correlated expression of *fos*^+^ cells between the BLA and MS (*Z*_obs_ = 2.53) as well as between the BLA and LS (*Z*_obs_ = 2.89) was greater in high E2 versus low E2 controls (indicated in blue). In contrast, correlations of the BLA and the PVT, a region involved in processing the experience of a prior stress (Bhatnagar and Dallman, 1998; Bhatnagar et al., 2003; Hsu et al., 2014) was reduced (*Z*_obs_ = −2.23, indicated in red).

Looking at the consequences of MAS on neuronal coactivation in high E2 mice (Fig. 7*B*) there was an increase in correlated *fos* expression between the BNST and the DG (*Z*_obs_ = 2.30), indicating altered coactivation of components of the extended amygdala and the hippocampal network. Coactivation was also increased between the MS and PVN (*Z*_obs_ = 2.47) and decreased between the MeA and PVN (*Z*_obs_ = −2.10) following MAS. Compared with the correlation of BLA and MS in high E2 controls compared with low E2 control mice (Fig. 7*A*), correlation between these two regions was reduced in high E2 MAS mice (*Z*_obs_ = −1.93). In contrast, the effects of MAS on patterns of coactivation in low E2 mice, which did not lose spatial memory following MAS, were distinct (Fig. 7*C*). Following MAS, there was an attenuation of coactivation between PVN and CA1 (*Z*_obs_ = −2.61), PVT and BLA (*Z*_obs_ = −2.14), and MeA and MS (*Z*_obs_ = −2.03). Coactivation of LS and PVT (*Z*_obs_ = 2.26) and MeA and DG (*Z*_obs_ = 2.19) was amplified after MAS. Notably, there was no alteration in correlation between BLA and MS (*Z*_obs_ = 0.42) following MAS in the low E2 mice.

These differential effects of MAS on high E2 versus low E2 mice culminated in disparate network connectivity patterns observed when comparing high and low E2 mice after MAS (Fig. 7*D*): The high E2 mice (with impaired spatial memory following MAS) had amplified coactivation between CA2/3 and BNST (*Z*_obs_ = 2.66), DG and BNST (*Z*_obs_ = 2.60), PVN and CA1 (*Z*_obs_ = 2.43), and LS and BNST (*Z*_obs_ = 2.05). Amplified coactivation between BNST and DG, the first node in the hippocampal trisynaptic pathway, and BNST and CA2/3, might indicate tight relation of salience/fear networks and the hippocampus, potentially intruding on and disrupting normal memory processes. Furthermore, there was attenuated coactivation between PVT and BNST (*Z*_obs_ = −1.98), LS and PVT (*Z*_obs_ = −2.39), MeA and CeA (*Z*_obs_ = −2.22), and MeA and DG (*Z*_obs_ = −1.96).

## Discussion

The principal findings of these experiments are as follows: (1) MAS impair spatial memory in female mice, as previously found for males, and this impairment depends on the phase of the estrous cycle. (2) Spatial memory is impaired by MAS in proestrous females, when physiological estradiol levels are high, but not during estrus, when estradiol levels are low. (3) Loss of hippocampus-dependent memory is accompanied by loss of dendritic spines, a proxy for excitatory synapses, in hippocampal CA1 of high-estrogen females only. (4) Activation of brain regions interconnected with the hippocampus, at basal conditions and following MAS, is modulated in a cycle-phase-dependent manner, suggesting a role for augmented network connectivity in the MAS-provoked memory disruption of high E2 females.

The present findings that higher levels of systemic estradiol in a female mouse predict stress-induced memory impairment were unexpected (Fig. 1). Estradiol is thought to be neuroprotective following stress or other neurologic disorders (Azcoitia et al., 2019). For example, repeated restraint stress impaired temporal order recognition memory in male rats, whereas female rats were protected. These differences were estrogen-dependent because blocking estrogen production or receptors during stress rendered females vulnerable and activating estrogen receptors in males protected their memory (Wei et al., 2014; Luine, 2016). *In vitro*, corticosterone may rapidly suppress NMDA-derived excitatory postsynaptic potentials in male hippocampal slices, but this suppression is abolished by estradiol (Ooishi et al., 2012). Notably, deleterious effects of higher estrogen levels during stress have been reported: high estrogen levels accelerated the acquisition of a conditioned response, but also provoked a more severe impairment following tail shock (Shors et al., 1998). Ovariectomy resulted in greater fear conditioning freezing behavior in females, and estradiol treatment reduced both contextual fear conditioning and hippocampal LTP (Gupta et al., 2001). Female rats in proestrus were more sensitive to an acute stress that impaired PFC-mediated spatial delayed alternation task compared with those in estrus (Shansky et al., 2006). Estradiol replacement in ovariectomized mice, although it increased contextual fear memory formation, reduced contextual fear extinction (McDermott et al., 2015). Together, these studies and others (Shansky et al., 2004, 2009) indicate that estrogen is not universally protective, and the interaction of stress and estrogen on memory is complex. Hormone levels, whether these were endogenous or exogenous, delivery regimen, period of deprivation, stressor type, memory task, time of day, and the underlying brain regions and networks are all crucial in interpreting the interactions between estrogen, stress, and memory (Holmes et al., 2002; McLaughlin et al., 2008; Barha et al., 2010; Babb et al., 2014; Korol and Pisani, 2015; Graham and Scott, 2018; Duong et al., 2020).

Here we examined the role of endogenous, physiological estradiol and the fluctuations of its levels throughout the estrous cycle in the effects of stress on memory. We first established the congruence of vaginal smears and estradiol levels (Fig. 2). Stress can alter cycle-dependent hormone fluctuations and estrous cycle duration (Galea et al., 1997; Shors et al., 1999; Liu et al., 2011; Wagenmaker and Moenter, 2017; Blume et al., 2019), but we excluded effects of the stressor used here on the duration of the estrous cycle. We focused on multiple acute concurrent stresses, such as those involved in mass shootings, assault, or natural disasters, events increasingly associated with the development of memory disorders. We have previously established that MAS-induced memory disruption and the associated spine collapse and synapse loss in males are attributed to the convergent activation of corticotropin releasing hormone receptor 1 and glucocorticoid receptor on dendritic spines (Chen et al., 2008, 2016). Downstream mechanisms of activation of both receptors converge on the RhoA-pCofilin signaling pathway (Chen et al., 2008). A role for estrogen in the effects of MAS on memory in males has not previously been suspected, although male hippocampal levels of estrogen are higher than those of proestrous females (Kato et al., 2013). The hormone is involved in memory processes and interacts with dendritic spines via the same RhoA-Cofilin pathway (Kramár et al., 2009a, 2009b, 2013; Chen et al., 2013). In the current study, MAS-induced memory impairment required high physiological estrogen levels in females (Fig. 1). This raises the possibility that, during cycle phases with high estradiol, activation of the classical stress-responsive receptors, glucocorticoid receptor and corticotropin releasing hormone receptor 1, is accompanied by synergistic engagement of estrogen receptors (ERα, ERβ, GPER, or a combination, within the hippocampus) (Mehra et al., 2005), to destabilize spines (Fig. 3), potentially via a converging signaling pathway. Additionally, high estrogen levels augment the stress response (Lo et al., 2000; Borrow and Handa, 2017; Heck and Handa, 2019). This amplified neuroendocrine stress response may contribute to the disruption of memory processes. Finally, while we selected mice in early proestrus when progesterone levels are low, a role of progesterone cannot be excluded. Progesterone has been demonstrated to counteract memory-promoting actions of estrogen (Chesler and Juraska, 2000; Harburger et al., 2007) as well as to modulate how stress impacts memory (Graham and Daher, 2016; Cohen et al., 2020). These hypotheses should be topics of future studies.

A need for afferent excitatory activation of the hippocampus for MAS-induced memory and dendritic spine loss is apparent from studies showing that corticotropin releasing hormone-induced spine loss requires glutamatergic receptor-mediated neuronal activation (Andres et al., 2013). Indeed, afferent regions identified here were differentially activated by MAS and cycle. MAS drove an increase in *fos* expression in the hypothalamic paraventricular nucleus in both cycle phases, but this activation was stronger in the high E2 mice (Fig. 4), perhaps suggesting augmented neuroendocrine stress response. The number of MAS-induced *fos*^+^ cells in the basolateral and medial amygdala, regions with monosynaptic connections to hippocampus (Pikkarainen et al., 1999; Petrovich et al., 2001; Beyeler et al., 2018), as well as the LS and the paraventricular nucleus of the thalamus, was selectivity increased in high estrogen females (Fig. 5). The BLA has been implicated in driving stress-induced hippocampal memory impairment (Rei et al., 2015) and the LS demonstrates a positive relationship with the intensity of a stressor (Úbeda-Contreras et al., 2018), suggesting that their projections to the hippocampus may contribute to loss of dendritic spines and memory function. These salient, hippocampus-projecting regions discussed above may work in concert to increase excitatory input to the hippocampus and promote spine diminution.

In males, MAS altered the cross-correlated activation between brain regions projecting to the hippocampus and between hippocampal subregions (Maras et al., 2014). Specifically, MAS, in contrast to a simple acute stress, reduced cross-correlation between the hippocampal formation and the septum and thalamus, regions involved in sensory processing, and enhanced coactivation of the hippocampus to the amygdala and extended amygdala, regions of the salience network. Using the same approach employed successfully by other groups (Wiersielis et al., 2016; Salvatore et al., 2018; Ruiz et al., 2020), we identified key differences between functional networks of high E2 and low E2 female mice both at baseline and in response to MAS. As previously established in males, augmented cross corelated activation of extended amygdala and the hippocampus was identified in high E2 MAS females (Fig. 6*D*). Before stress, high E2 mice had greater correlation of *fos* expression between the BLA and MS compared with low E2 controls (Fig. 7*A*). The BLA and MS are thought to drive excitation in the hippocampus in a coordinated manner (Spanis et al., 1999; Bergado et al., 2007), which might support hippocampal memory. We found that, in high E2 females after MAS, correlated activity between the BLA and MS was diminished (Fig. 7*B*), potentially reflecting disruption of network function supporting memory. As mentioned, hippocampal estradiol levels are generally higher in both males and proestrous females, consistent with the notion that estradiol may influence functional network activity, acting at the circuit levels in addition to actions at receptors on individual dendritic spines.

In conclusion, the current studies describe an important correlation of high physiological levels of estradiol to stress-induced memory impairments. This potentially deleterious role of estradiol is novel and underscores the need for careful and nuanced studies of the role of sex and sex steroids on the effects of distinct stresses in distinct contexts (Simmons et al., 2020). Such studies and an improved understanding of the underlying mechanism are a prerequisite for elucidating the biology underlying sex differences in post-traumatic stress disorder and other stress and memory-related disorders.
